# “Up to and including death”: understanding anaesthesia‐related anxiety among pet owners in a tertiary‐care setting

**DOI:** 10.1111/jsap.70168

**Published:** 2026-07-11

**Authors:** A. Shuttleworth, C. Adami

**Affiliations:** ^1^ Department of Veterinary Medicine University of Cambridge Cambridge UK

## Abstract

**Objectives:**

Preparing clients for their pet to undergo general anaesthesia is an important and common task for veterinary professionals. The aims of this study were to better understand referral client anxiety relating to general anaesthesia and to determine their level of knowledge in this area.

**Materials and Methods:**

Pet owners attending a UK referral centre whose dog or cat had undergone general anaesthesia between February 6, 2022 and February 6, 2025 were invited to take part in an online survey. The questionnaire comprised four sections: demographics, concerns regarding general anaesthesia, knowledge of this area and provision of consent.

**Results:**

In total, 432 responses were included in analysis. 69.2% of respondents reported feeling “anxious” towards general anaesthesia, with 60.9% indicating that they had concerns. Of the categories of concern reported, anaesthetic death and contextual factors were cited in 31% and 30% of responses, respectively. The mean average mortality rate estimated by respondents for healthy dogs and cats was 16.2% (95% confidence interval: dogs 14.20 to 18.25, cats 14.01 to 18.35) compared to 9.7% (95% confidence interval: 8.17 to 11.26) for humans. 89.4% of respondents were happy with the level of information they received ahead of the anaesthetic and 88.5% reported that they provided informed consent. When provided with published statistics for anaesthetic mortality in UK dogs and cats, 63% of participants found this reassuring.

**Clinical Significance:**

Causes of owner anxiety regarding general anaesthesia are multifactorial and do not always relate to mortality. UK referral clients may perceive a higher rate of anaesthetic mortality in healthy dogs and cats than currently evidenced.

## INTRODUCTION

Dogs and cats commonly undergo general anaesthesia (GA) in veterinary practice and preparing owners ahead of this is a common task for veterinary staff. Providing information, addressing concerns and obtaining informed consent are key features of these consultations. However, to date, there is very little literature within the veterinary field relating to pet owners' understanding of anaesthesia and their concerns surrounding it.

In the human healthcare field, efforts have been made to standardise pre‐anaesthetic information, including complication rates, provided to patients and this is available in a variety of multimedia formats (NHS, 2024, RCoA, 2024). Along with this, there is a growing body of evidence supporting the beneficial effect of pre‐anaesthetic consultation on patient satisfaction, anxiety and mortality (Ambulkar et al., 2022; Blitz et al., 2016). A link has been identified between patients who report being dissatisfied with the level of pre‐anaesthetic information they have received and their level of anxiety relating to anaesthesia (Bello et al., 2023). Research into the optimal timing and format in which this information should be conveyed to patients is ongoing. Jlala et al. (2010) found that use of a short preoperative film reduced patient anxiety immediately before surgery and postoperatively, advocating for the use of such audiovisual means of conveyance. Similarly, a systematic review in this area supported the notion that information provided in multiple formats improved patient knowledge (Hounsome et al., 2017). However, the same review found no conclusive evidence to promote specific timings or formats that improved patient anxiety.

An area in which parallels may be drawn between human and veterinary medicine is the delivery of information to the consenting party, separate to the patient undergoing anaesthesia. Though the parent–child bond is significantly different to that between a pet and their owner, there are similarities that may be applied between the two when considering the pre‐anaesthetic consultation. Currently, there is little research evaluating the information that pet owners desire ahead of GA or indeed their level of knowledge relating to anaesthetic risk.

There is a paucity of information regarding what specifically concerns pet owners regarding GA and though many may assume the main fear would be anaesthetic death, we have no evidence to support this assumption at present. Anaesthetic death has been identified as the greatest concern for human patients irrespective of type of surgery undertaken (Burkle et al., 2014). A later study by Bello et al. (2023) also found anaesthetic mortality to be the most commonly reported fear of adult patients, followed by postoperative nausea and vomiting. Conversely, the highest ranking concern among parents of children undergoing GA was postoperative pain (Wisselo et al., 2004). Whether pet owners attending a tertiary‐care facility would more closely match patients, parents or have an entirely different list of concerns remains to be elucidated.

This study aimed to determine the level of anaesthesia‐related anxiety experienced by pet owners at tertiary‐care level, along with their awareness and perception of mortality risk and desire for information. We hypothesised that respondents would report high levels of anxiety relating to GA and that anaesthetic mortality would be the most common concern raised.

## MATERIALS AND METHODS

A questionnaire was designed based around four key areas: demographics, attitude towards anaesthesia, knowledge relating to anaesthesia and informed consent. The Checklist for Reporting Results of Internet *E*‐Surveys (CHERRIES) guidelines assisted in the formation of this questionnaire (Eysenbach, 2004).

The questionnaire consisted of four sections: demographics, concerns regarding GA, knowledge of veterinary GA and providing consent for GA.

Demographic questions related to respondent age, gender, occupation and highest level of qualification. Information relating to their pet was also obtained at this stage, including species (dog or cat), subjective state of health at the time of GA, hospital service visited, nature of procedure and timeframe between recommendation of the procedure and its commencement. All questions in this section were multiple choice allowing a single answer.

In the attitudes towards anaesthesia section, respondents were first asked how they felt ahead of their pet's GA to which four options were available: terrified, anxious, indifferent or excited. This section also provided respondents with the opportunity to list their concerns ahead of their pet's GA in a free‐text format.

The third section obtained information regarding owner knowledge of veterinary GA *via* a multiple‐choice question allowing more than one answer (who could perform their pet's GA) and free‐text entry (estimated mortality rates for healthy dogs, cats and humans).

The final section, relating to informed consent, collected information regarding the quantity and type of information owners wanted before their pet underwent GA. Single best answer multiple choice questions were used to determine if owners had a desire for further information, how they would like to receive this and if they felt they had provided informed consent. Free‐text entry boxes enabled owners to list the exact information that they desired. Finally, owners were provided with published statistics relating to anaesthetic mortality for healthy dogs and cats in the UK and asked whether knowing this ahead of time would have impacted their level of reassurance (Brodbelt, 2009; Shoop‐Worrall et al., 2022).

Prior to launching the online questionnaire, it was trailed in‐person with eight registered clients whose dog or cat had recently undergone GA and were re‐presenting to the hospital for a follow‐up appointment. Responses to these questions were not included in the overall results of this study and instead were used to adapt question structure, response options and accessibility of the questionnaire. No questions were added or removed following the pilot interviews.

The online questionnaire was constructed using Qualtrics survey software (Qualtrics, Provo, UT, accessible *via* www.qualtrics.com). Email addresses of clients attending The Queen's Veterinary School Hospital whose dog or cat had undergone GA between February 6, 2022 and February 6, 2025 were obtained from the hospital's database. Only the email addresses of clients who had consented to be contacted in relation to research were used. This yielded a list of 3111 email addresses, all of which were contacted with an invitation to complete the online questionnaire. The questionnaire was live for 1 month, May 1, 2025 to May 31, 2025. On accessing the survey, respondents were provided with a background of the study and made aware that participation was voluntary with no incentive offered. Owners were only able to access the body of questionnaire if they provided informed consent to use of their data on the first screen. Recipients were only able to complete the questionnaire once and no identifiable information was obtained during the course of the study.

The questionnaire can be viewed in its completeness in Supporting Information Appendix I.

Ethical approval for this study was granted by the Human Biology Research Ethics Committee of the University of Cambridge (reference HBREC.2024.18).

### Statistical analysis

Participation rate was calculated as the number of pet owners who received the online survey by the number of those who opened the link and started it. The completion rate was determined by dividing the number of participants who fully completed the questionnaire by the number who accessed it but failed to submit a complete response. Responses in which less than 50% of the questionnaire were completed were excluded from data analysis.

Descriptive statistics and analysis of proportions were used. The chi‐squared test of independence was used in conjunction with contingency tables and degrees of freedom to investigate associations between categorical variables (NIST/SEMATECH, 2012). For this purpose, the variable “profession” was categorised into a dichotomy outcome (“animal or human healthcare professionals” *vs*. “others”), while the perceived risk of death, provided by the participants as free text, was categorised as 3% to 5%, 6% to 10%, and >10%.

Commercially available statistical software was used (SigmaStat 3.5 and SigmaPlot 10, Systat; SPSS, version 28, IMB Corp.). Statistical significance was set at values of *P* < .005. Graphs were created using Microsoft Excel®, Microsoft Corporation, 2024.

## RESULTS

### Demographics

A total of 486 responses were received to the online questionnaire, producing a participation rate of 15.6% (486/3111). Ten respondents failed to consent to use of their data and were excluded from analysis (2.3%, 10/486). Three hundred and eighty‐nine respondents completed the entire questionnaire, resulting in a completion rate of 80% (389/486) and a response rate of 12.5% (389/3111).

Eighty‐seven responses were incomplete, defined as those which had completed less than 100% of the questionnaire (17.9%, 87/486). Forty‐four respondents completed less than 50% of the questionnaire and were therefore excluded. The final number of responses included in analysis was 432. Fig 1 contains a schematic representation of how responses were evaluated for inclusion.

**FIG 1:**
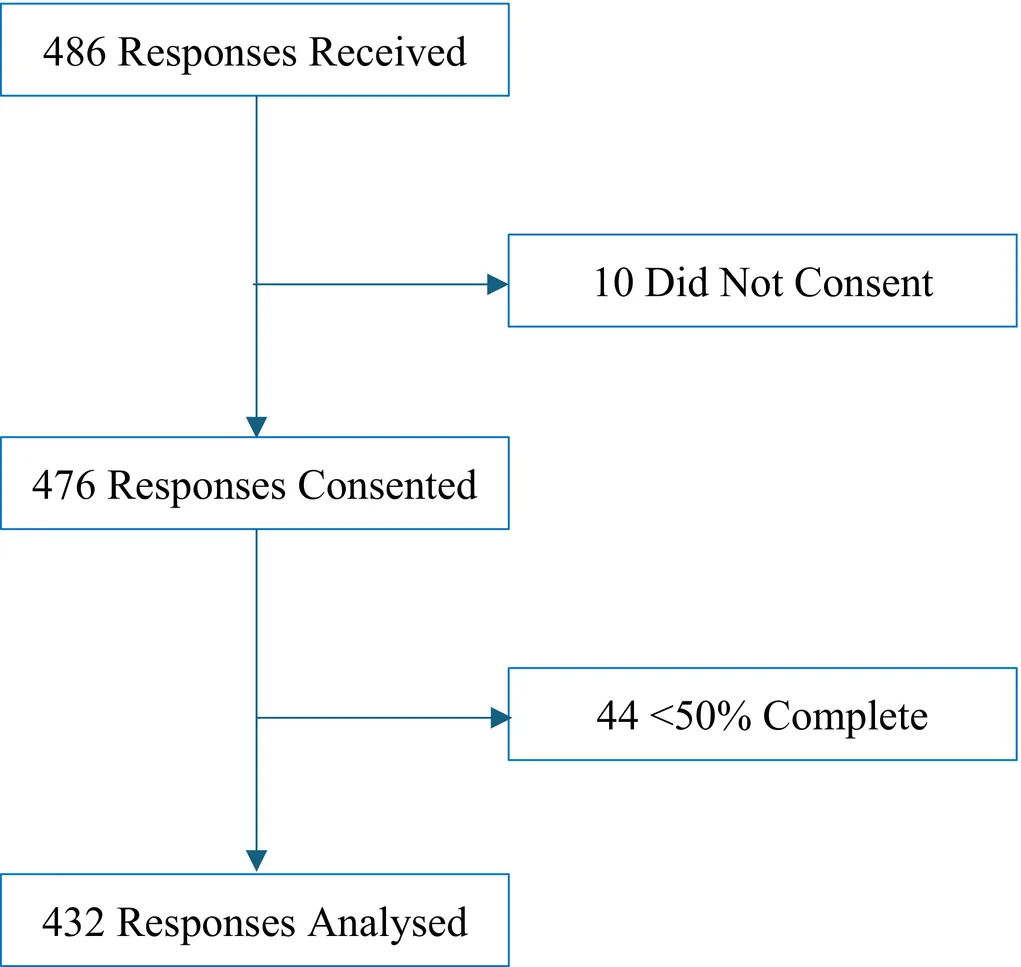
A schematic representation of the process by which responses to an online survey of pet owners attending a UK referral centre were included or excluded for analysis.

Demographic results are listed in Table 1. Responses were obtained from all age brackets, with the highest number received from those between 50 and 59 years of age (29.4%, 127/432). A greater proportion of respondents identifying as female (78.5%, 339/432) completed this questionnaire. All response options for field of work were represented, with “other” being the most frequently selected option (20%, 86/432). While responses across all qualifications were obtained, the highest number of responses reported a bachelor's degree to be their highest level of qualification (25.2%, 109/432). Both dog and cat owners completed this questionnaire, though the majority indicated that they were completing this questionnaire in relation to their experience with a pet dog (81.9%, 354/432).

**Table 1:** Demographic responses of 432 referral clients participating in a survey of attitudes towards general anaesthesia

Demographic	Category	Count (*n*)	Percentage (of 432)
Age bracket	20 to 29	12	2.8
	30 to 39	44	10.2
	40 to 49	75	17.4
	50 to 59	127	29.4
	60 to 69	109	25.2
	70 to 79	51	11.8
	>80	9	2.1
	Prefer not to say	5	1.2
Gender	Male	87	20.1
	Female	339	78.5
	Non‐binary	2	0.5
	Other	1	0.2
	Prefer not to say	3	0.7
Field of work	Agriculture	5	1.2
	Animal care (including veterinary medicine)	36	8.3
	Art & entertainment	9	2.1
	Education & academia	47	10.9
	Environment & energy	2	0.5
	Hospitality	8	1.9
	Human healthcare & social work	58	13.4
	Information technology	33	7.6
	Law	16	3.7
	Leisure & tourism	4	0.9
	Manufacturing & construction	16	3.7
	Media & marketing	7	1.6
	Public services	22	5.1
	Retail & sales	19	4.4
	Science (including pharmaceuticals & engineering)	20	4.6
	Transport	3	0.7
	Other (please specify)	86	19.9
	Not applicable	33	7.6
	Prefer not to say	8	1.9
Highest qualification	GCSE/O‐level	78	18.1
	A‐level, BTEC or equivalent	80	18.5
	Apprenticeship	10	2.3
	Foundation degree	13	3.0
	Bachelor's degree (BSc or BA)	109	25.2
	Master's degree (MSc or MA)	65	15.0
	Doctor of philosophy (PhD) or other doctorate	19	4.4
	Other (please specify)	37	8.6
	Prefer not to say	21	4.9
Pet species	Dog	354	81.9
	Cat	78	18.1

### Concerns regarding anaesthesia

When asked for their subjective perception of their pet's state of health ahead of undergoing GA, the majority of respondents indicated that their pet was either “moderately unwell” (29.2%, 126/432) or “severely unwell” (28.2%, 122/432). 21.2% (92/432) of owners reported that their pet was healthy at the time of their GA. The most frequently cited services being visited by the 432 respondents were soft tissue surgery (23.4%, 101/432), neurology (21.1%, 91/432) and oncology (16.7%, 72/432). 14.4% (62/432) of owners reported that they did not know which service they visited. When asked for the nature of their pet's procedure, 44.7% (193/432) selected “diagnostic investigations”, 25.9% (112/432) selected “non‐elective or emergency surgery” and 22.2% (96/432) selected elective surgery. Eight respondents failed to answer question nine regarding the timeframe between recommendation of their pet's procedure and its commencement, resulting in a total of 424 responses for analysis. The most selected option was “less than 24 hours” (31.1%, 132/424) followed by “over 24 hours but less than 1 week” (28.8%, 122/424) and “over 1 week but less than 1 month” (27.4%, 116/424).

Fig 2 demonstrates that the majority (69.2%, 299/432) of owners reported feeling “anxious” regarding their pet's anaesthetic, while 10.2% (44/432) indicated that they were “terrified”.

**FIG 2:**
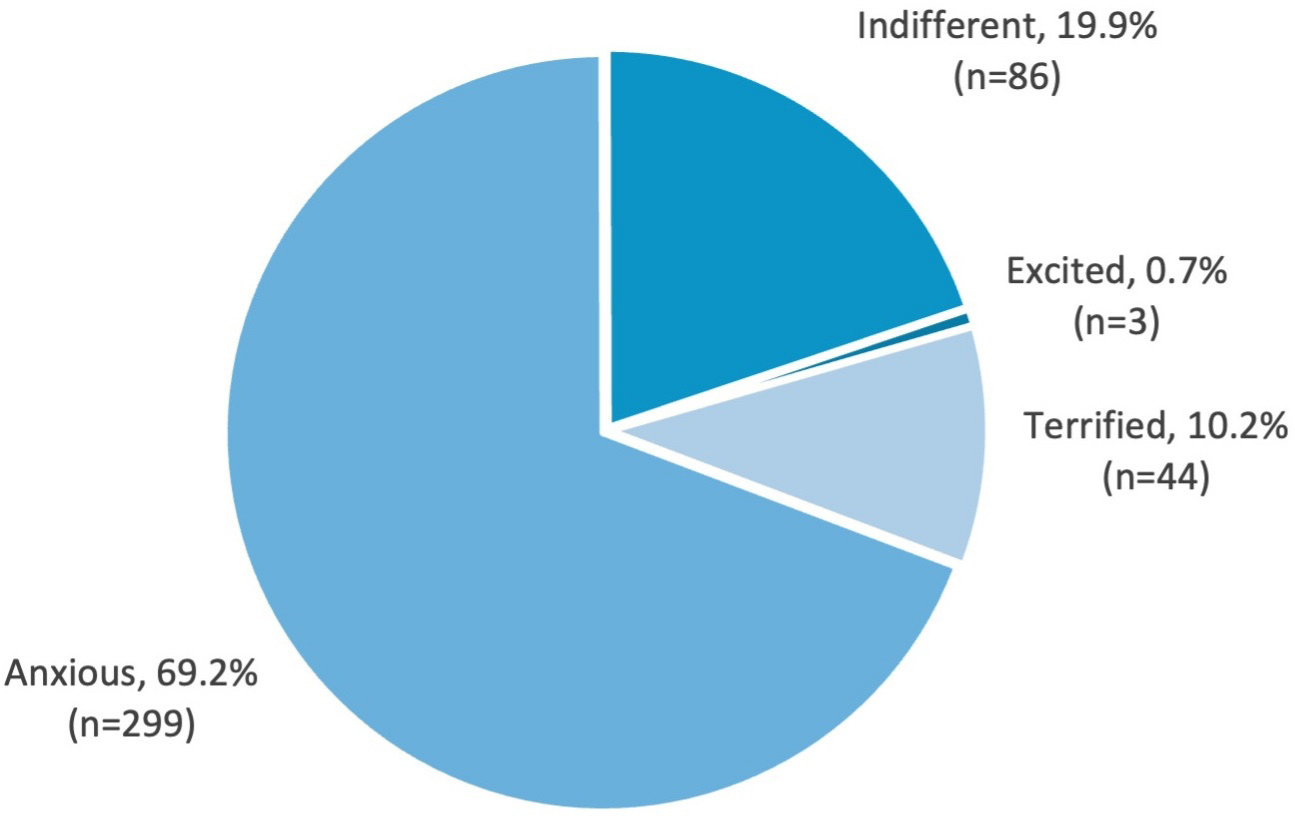
Referral client responses when asked to indicate their feeling towards their pet's general anaesthetic.

When owners were asked if they had concerns regarding their pet's GA, 60.9% (263/432) answered “yes” and 250 of these respondents went on to specify their concerns. These 250 responses generated a list of 309 concerns (as some responses contained multiple) in free‐text format. On examination of these responses, the authors identified recurrent themes of concerns that went on to form categories to facilitate analysis. The following categories were identified: Anaesthetic death; Complications and adverse events associated with the anaesthetic or procedure; Hospitalisation (including perceived stress to the animal and behaviour towards staff); Undergoing multiple GAs in a short period; Contextual factors (specific concerns relating to age, comorbidities or breed); Recovery from GA; and Non‐specific concerns.

A list of key words relating to each category was generated and a search function was employed using these key words to categorise each response. Where multiple key words were included, the response was counted in each category once. If the response contained no key words or relevance to categories 1 to 6, it was allocated to “non‐specific concerns”.

Table 2 lists the key words used for categorisation along with examples of comment allocation. Fig 3 displays the categories of concerns reported as a percentage of total concerns.

**Table 2:** Categorisation of respondent concerns regarding general anaesthesia using keywords along with example responses from each category

Category (keywords)	Example responses
Anaesthetic death (die, death, dying, would not wake up/make it/come round, survive, lost, life)	*That she may die under the anaesthetic* *Our dog wouldn't make it* *I've lost 3 animals to anaesthetic before so this was at the forefront of my mind*
Complications and adverse events (complication, adverse event, reaction, react, outcome)	*Sedation makes him symptoms worse usually so I was a bit anxious in case it was triggered* *Not being able to metabolise the medication properly and having complications* *Whether she would have any adverse reaction*
Hospitalisation (hospital, hospitalisation, kennel, anxiety, stress, stressed, behaviour, nervous, leave, fear, separation, not see him/her/them, leave him/her/them)	*How traumatic the experience would be for my dog* *About managing her anxiety leaving her in a strange place* *[…] that the dog would not be stressed nor frightened by experience*
Multiple anaesthetics (multiple anaesthetics/procedures, repeat, repeated)	*[…] having general anaesthetics [especially repeated ones] was not good for patients* *He had already had an anaesthetic a few days before […]* *[…] would he be ok with the number of times he would need to go under for radiotherapy*
Contextual factors (age, years, breed, brachycephalic, heart, murmur, lung, neurological, seizure, kidney, renal, livery, hepatic, disease, unwell, illness)	*Our dog is brachycephalic so the concerns are related to the increased risk associated with this type of dog […]* *He was 12 years old and I believe that there are increased risks to an anaesthetic when you are older* *As the cat had a heart murmur, the concern was how his heart would cope with anaesthesia*
Recovery (recovery, coming round, waking up)	*Recovery from potentially a long time under anaesthetic* *My dog always struggles to come round after even a small amount of anaesthesia* *Would she come round ok*
Non‐specific concerns	*General concern for dogs well‐being* *The usual concerns regarding a general anaesthetic* *Whether we were doing the right thing*

**FIG 3:**
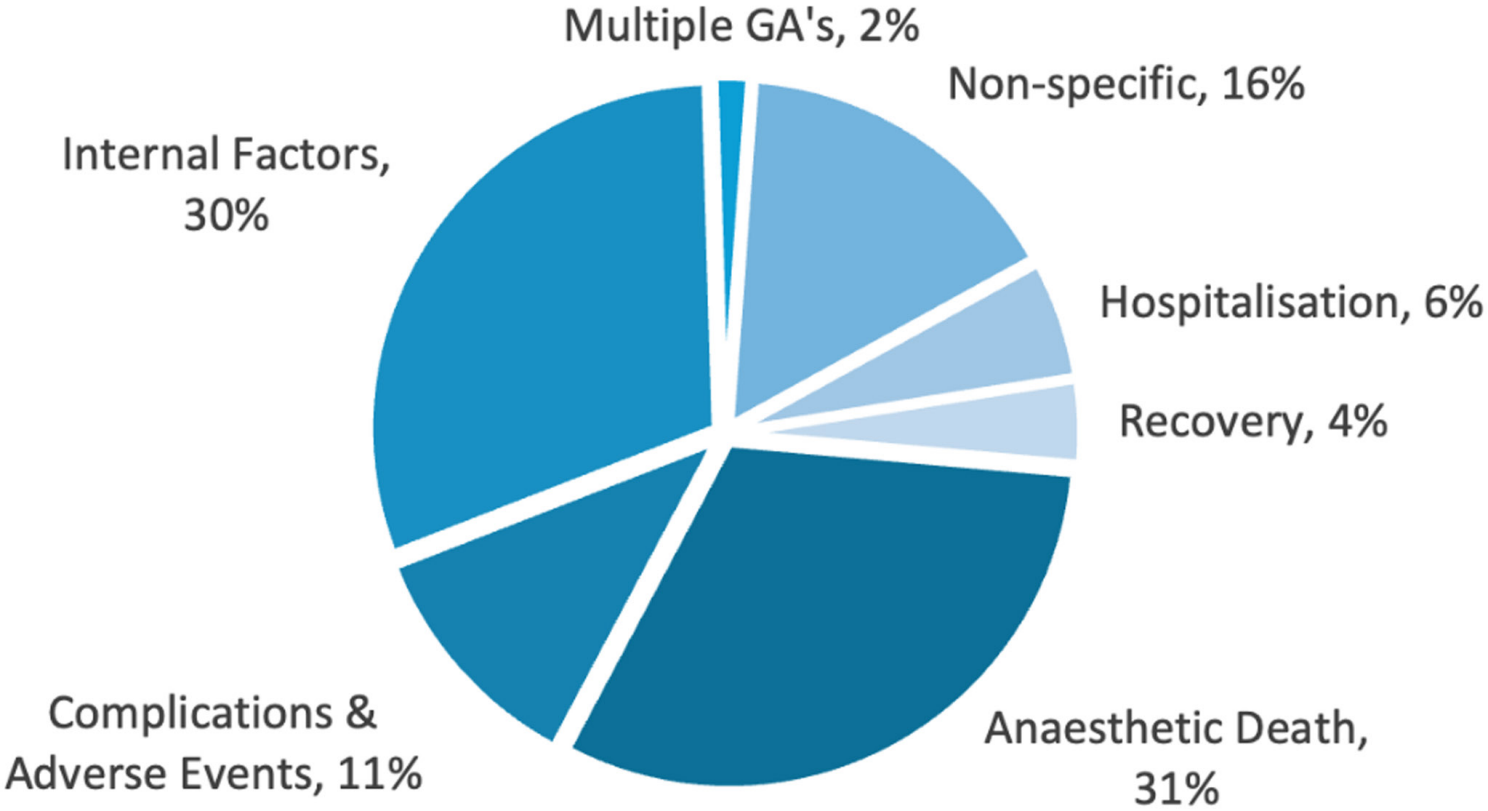
Categories of concern reported by pet owners attending a UK referral centre regarding general anaesthesia.

A total of 418 owners provided a response when asked if their pet had undergone GA before at any centre. Of these owners, 72.5% (303/418) answered “yes”, 24.6% (103/418) answered “no” and 2.9% (12/418) were unsure.

### Knowledge of veterinary anaesthesia

When asked who they thought could perform their pet's GA at a referral centre, 411 owners gave a response. As multiple answers could be chosen, the total number of selections was 725. “A veterinary surgeon with advanced qualification(s) in anaesthesia” was the most frequent response, representing 50% of total selections (363/725). Fig 4 displays the frequency of selection for all available response options.

**FIG 4:**
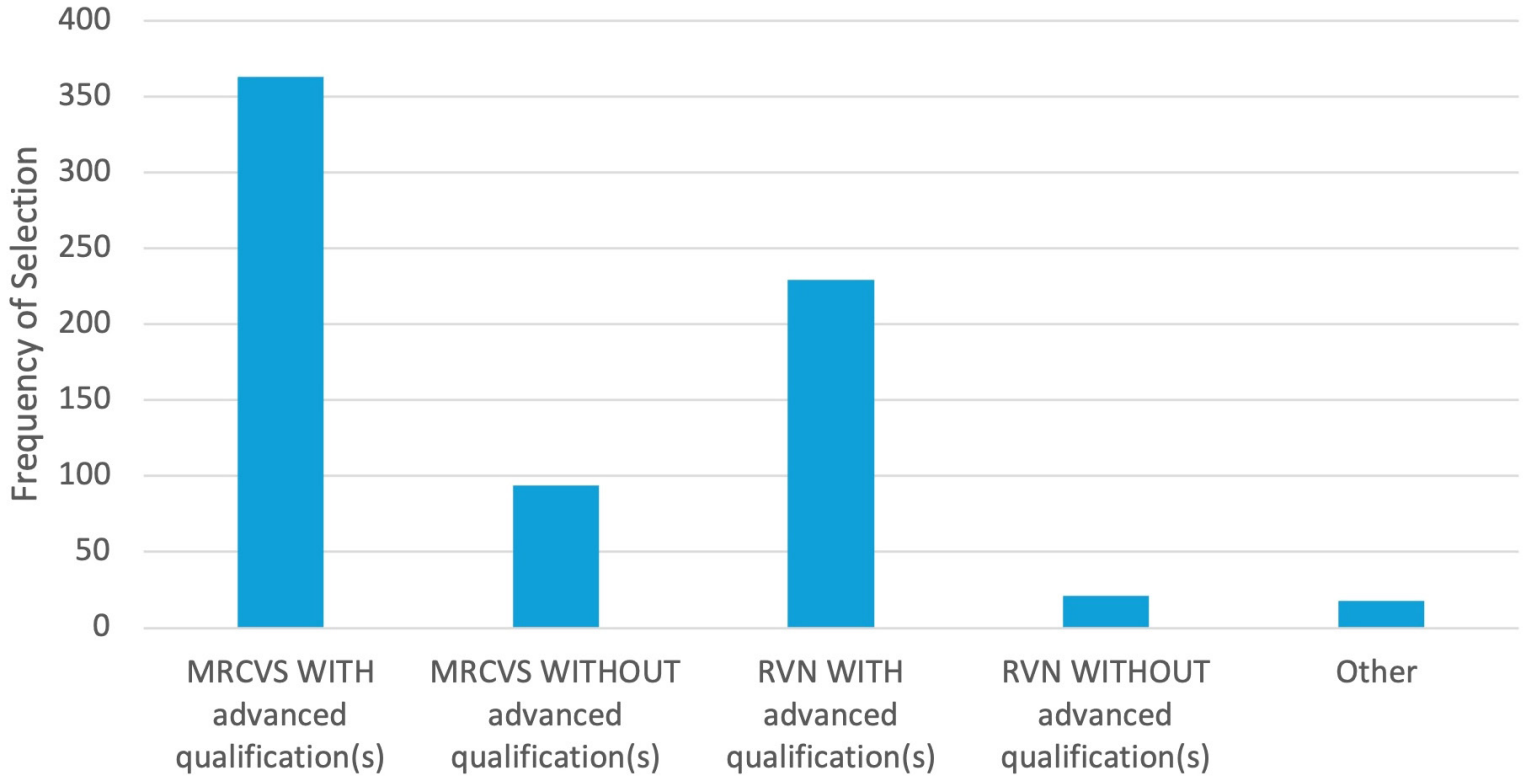
Responses of 411 pet owners when asked who may perform their pet's general anaesthetic at a referral hospital.

The number of respondents providing an answer for what they believed to be the risk of anaesthetic mortality for a healthy patient varied with species. Respondents estimated a significantly lower mean mortality risk for humans (mean: 9.7%, 95% confidence interval: 8.17 to 11.26; *n* = 373) compared with dogs (mean: 16.2%, 95% confidence interval: 14.20 to 18.25; *n* = 354) and cats (mean: 16.2%, 95% confidence interval: 14.01 to 18.35; *n* = 288) (*P* = .001).

Three hundred and ninety‐six answers were obtained to the question of whether respondents had received all of the information they wanted ahead of their pet's GA, with 89.4% (354/396) answering “yes”. Of the 42 owners responding “no”, 39 indicated what further information they would have liked. Respondents answered in their own words, with most answers relating to specific risks associated with GA and more detail relating to the practicalities of the procedure and anaesthetic. A further 38 respondents went on to specify how they would like to receive such information, with 39.5% (15/38) requesting a consultation with the anaesthetist, 31.6% (12/38) wanting a consultation with the procedural vet, 13.2% (5/38) satisfied with a leaflet or letter and 15.8% (6/38) selecting “other”.

### Providing consent for GA

When asked if they had received enough information to provide informed consent for GA, 391 responses were obtained. 88.5% (346/391) selected “yes”, 2.3% (9/391) selected “no” and 9.2% (36/391) indicated that they were “unsure”.

Finally, when provided with reported statistics of anaesthetic mortality in dogs, cats and humans, 389 owners went on to indicate whether this had impacted their level of reassurance. 63% (245/389) were “more reassured”, 36.2% (141/389) reported feeling “the same” and 0.8% (3/389) indicated that they then felt “less reassured” (Fig 5).

**FIG 5:**
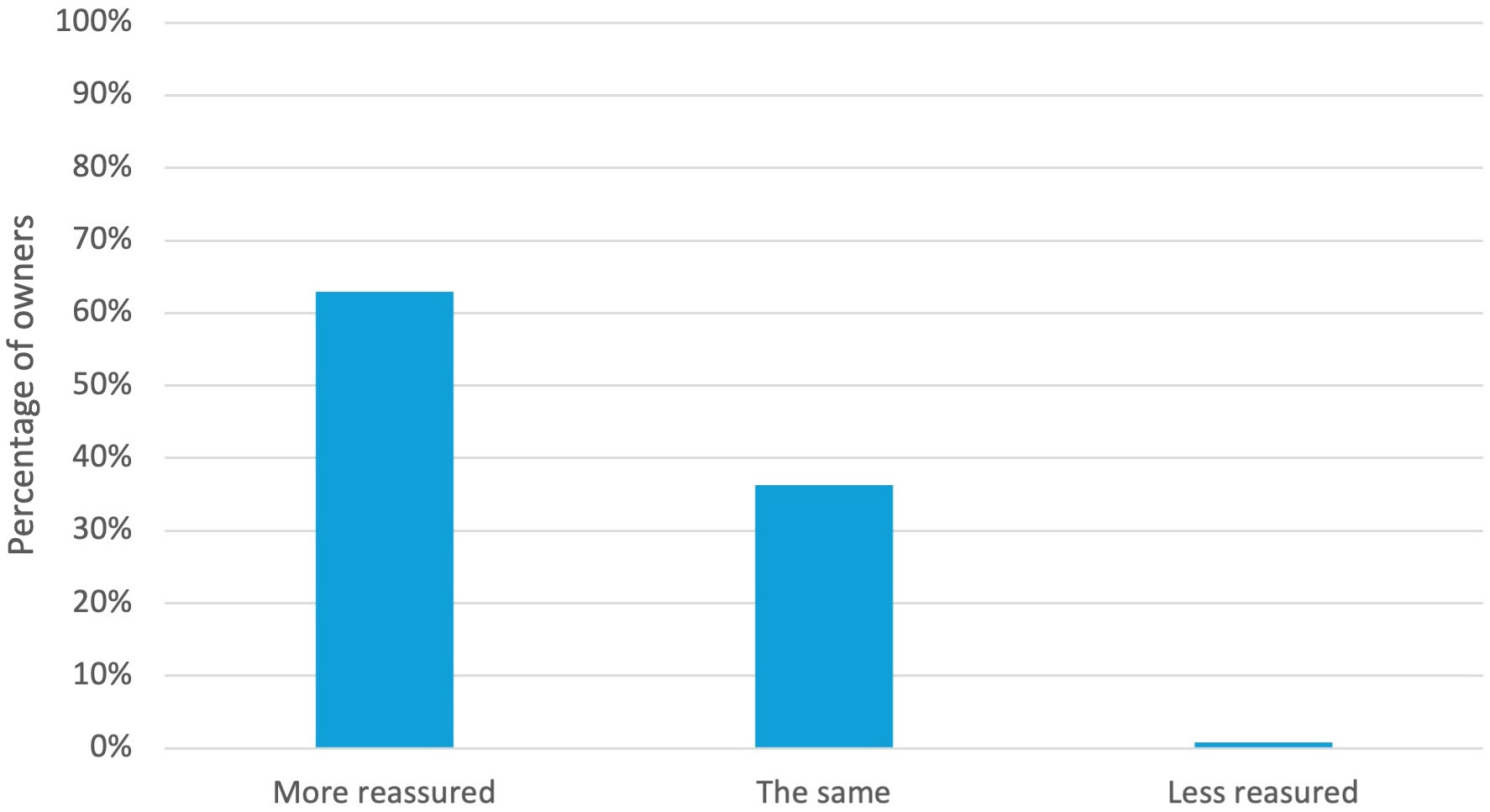
Responses from 389 referral clients when asked how current statistics regarding anaesthetic mortality rates for healthy dogs and cats impacted their level of reassurance relating to general anaesthesia.

## DISCUSSION

The responses to this questionnaire have provided an insight into the awareness and understanding that referral clients possess when presenting their dog or cat to undergo GA. The results indicate that most owners feel anxious regarding GA, with the majority citing concerns relating to mortality or factors relating to age, breed or current comorbidities. This anxiety may be partially explained by the high rates of anaesthetic mortality in healthy dogs and cats perceived by owners. Providing owners with published statistics regarding mortality may offer some reassurance in this regard.

Demographic results obtained across all categories indicate that this questionnaire received responses from a representative sample of referral clients, though the majority identified themselves as dog owners. This may reflect the greater attendance of dogs at referral centres in the UK or more specifically at the centre hosting this study (Corr et al., 2024). There was a relatively even spread across the state of health categories, though this did require the respondent to self‐assess their pet's health state and therefore may not coincide with that determined by a veterinary professional. While responses were received from pet owners visiting all available disciplines, 14.4% of respondents were unable to indicate any discipline that they had contact with during their visit to the hospital. This may be due to failing recollection or may indicate that some owners are not fully aware of the service treating their pet and may benefit from clearer communication at both the hospital and referring practice. No statistically significant relationships were identified between any of the demographic or attitude variables and how respondents felt ahead of the GA. The implication here may be that a pet undergoing GA is likely to produce anxiety in the majority of owners, independent of their background.

As hypothesised, referral clients most commonly felt anxious regarding GA with anaesthetic death the single most cited category of concern. However, when considering all categories reported, 69% related to factors other than anaesthetic death. These other concerns may be easier to rationalise and alleviate compared to the, unfortunately, ever‐present risk of death. While not possible to definitively conclude based on the presented data, spending more time counselling referral clients regarding these non‐mortality‐related concerns may improve levels of anxiety around GA. The high frequency of mortality concerns is consistent with most common fears of adult human patients but differs to those of parents whose child was undergoing GA (Bello et al., 2023; Burkle et al., 2014; Wisselo et al., 2004). Interestingly, no respondent listed pain as a concern, conflicting with a study of parents whose child was undergoing GA, in which pain was the most frequently reported concern (Wisselo et al., 2004). This raises numerous questions, specifically did respondents consider pain as a factor but not find it concerning, or was the potential for pain not deemed likely? It is possible that the referral clients surveyed were able to consider their concerns entirely in the context of GA and eliminate those that may relate to the procedure that the GA facilitates. The impact of the human–animal bond and the altered dynamics of a caregiver–animal relationship make direct comparison with human medicine challenging.

In this study, the mean mortality rate estimated by referral clients presenting their pet for GA was 16.2%, which is considerably higher than the 0.009% to 0.11% reported in the literature (Brodbelt, 2009; Shoop‐Worrall et al., 2022). The reasoning behind this perceived higher rate remains unclear and could relate to numerous factors, such as individual experiences, lack of medical knowledge or emphasis placed on death as a risk factor during the pre‐anaesthetic consultation. The referral hospital setting may have also impacted the estimated mortality rate, as clients presenting their pet for a routine elective procedure may perceive a lower risk than those whose pet is undergoing a complex procedure with advanced disease. In the authors' experience, many anaesthetic consent forms provide little detail of complications beyond mortality, and in busy practices with limited consultation time, there may not be the opportunity to discuss these in any great detail. This leaves the consenting client at risk of developing anchoring bias, in which they may place too great an importance upon the first, or often only, complication listed (Ly et al., 2023). If a client is first told that every GA carries a risk of death, it stands to reason that they may fixate upon this, especially if no risk contextualisation is offered simultaneously. Providing a prescriptive list of complications to be discussed for every patient in every pre‐anaesthetic consultation is both impossible and beyond the scope of this study. However, including the more common adverse events, such as hypotension, bradycardia, hypoventilation and hypothermia, may offer referral clients a more realistic appreciation of anaesthetic risk (Brown & Murison, 2024; Bruniges & Rioja, 2019; Redondo et al., 2007). The Royal College of Veterinary Surgeons offers guidance on gaining informed consent for veterinary procedures and advises that a key part of this is conveyance of the main risks associated (RCVS, 2024). As such, it seems advisable that when discussing anaesthetic risks with clients, more time should be spent detailing these more likely events and their mitigation rather than exclusively dealing with mortality. When designing anaesthetic consent forms, it may be prudent to avoid listing solely death as a potential complication or glossing over other potential risks with phrases such as “complications up to and including death” or “anaesthesia carries a risk to life”. Reassuringly, in the present study, the majority of respondents indicated that they had all the information necessary to provide informed consent. However, the uncertainty or lack of informed consent reported by the remaining 11.5% of respondents should give veterinary surgeons pause for thought. Clear communication is imperative when requesting consent and owners must be given the opportunity to ask questions and withhold consent if they feel this is appropriate.

Responses to this survey indicate that owners were largely satisfied that they had received all of the information they desired ahead of GA, implying sufficient information was delivered during the pre‐anaesthetic consultation. However, there remains the possibility that anxious pet owners may rely on information avoidance as a coping strategy, impacting their response to this question. When determining the level of information that should be conveyed to patients, parents or pet owners, it is important to consider that people usually adopt one of two coping styles, monitors or blunters (Miller & Mangan, 1983). Monitors desire a high level of information and lack of it may lead to anxiety, while the opposite is true for blunters. Interestingly, Wisselo et al. (2004) found no impact of parental coping style on the level or type of information desired ahead of their child's GA, suggesting caregivers may change their information‐seeking behaviour when it regards their dependent. Where further information is desired, it appears that having the option of a consultation with an anaesthetist may be preferable to some owners, while others may be satisfied with a material resource. A systematic review of anaesthetist–patient communication from the human field highlighted that patients are largely satisfied with such interactions and that discussion of risk in this context does not usually lead to distress (Tylee et al., 2020). Where desirable, the opportunity to discuss their pet's GA with an anaesthetist may be valuable to referral clients and may have a positive impact on their feelings towards GA.

Another important factor to consider is the timing of information delivery on client recollection. A balance must be struck between providing clients with time to consider the information conveyed and the potential for failure of recollection. Gillies et al. (2013) found that when provided with information relating to anaesthetic risk 2 weeks before GA, most patients could not recall all of the complications listed when asked on the day of surgery. Moreover, these authors identified that 26% of patients were unable to recall a single complication provided. As such, it seems prudent to provide information at multiple timepoints and in both verbal and written format, with the intention of enhancing client recollection.

Brodbelt (2009) previously found that the risk of anaesthetic mortality was significantly higher in cats than in dogs, especially in healthy animals. However, more recent reports have found that the worldwide mortality rate in cats is similar to that of dogs, 0.63% and 0.69% respectively (Redondo et al., 2023, 2024). Interestingly, respondents to this study generally estimated the risk of mortality to be the same for cats and dogs, though much higher than published mortality rates. Owners were correct in their estimations that the risk of anaesthetic death in healthy humans (0.001%) is lower than that of dogs and cats, but still considered this to be much higher than the rate reported (Brodbelt, 2009; RCoA, 2023; Shoop‐Worrall et al., 2022). Overall, it can be appreciated that referral clients believe GA to be a higher‐risk procedure than veterinary professionals and it is therefore appropriate to offer judicious reassurance during pre‐anaesthetic consultations. Involvement of an anaesthetist in the pre‐anaesthetic consultation could reduce owner anxiety regarding GA but may also enhance the value of care perceived by owners and highlight the role of this somewhat hidden discipline (Mitek et al., 2019). While owners did not unanimously agree that knowledge of mortality statistics was reassuring, it appears that only a small minority of respondents found this unhelpful. For owners who appreciate tangible evidence for the safety of GA, providing up‐to‐date mortality rates may be of benefit.

This study faces multiple limitations. Firstly, the response rate was suboptimal when compared to the average within the human healthcare sector, despite implementation of multiple reminder emails (Meyer et al., 2022). This was likely impacted by the extended time period for inclusion, meaning that clients who had visited the hospital multiple years prior were invited to participate but may not have responded due to limited recollection. Demographic results indicate that respondents who were dog owners, female and over the age of 50 were highly represented, impacting the generalisability of any conclusions drawn. There was a general lack of statistically significant findings in this study, potentially relating to the descriptive nature of the results obtained. Including a large number of response options may have decreased statistical significance in this study; however, overreliance on categorisation would likely have yielded less detailed information. This study did not define anxiety or its related terminology, and no validated scoring system was introduced; instead, respondents were required to self‐report their level of anxiety. The subjectivity of the language used creates the potential for individual interpretation, and therefore inter‐respondent variability in the boundaries between each level of anxiety. Inclusion of a validated scoring system for anxiety in any future in this area may enhance objectivity and reliability.

As previously stated, pre‐anaesthetic consultation is not standardised and therefore the single‐centre results presented here may not be representative of all veterinary hospitals. Use of a tertiary‐care centre may have exposed this study to referral filter bias, whereby owners of pets with more complex or severe disease may have differing anxiety and a disparate list of concerns regarding GA compared to a primary‐care population (Sackett, 1979). Furthermore, the connotations and expectations that pet owners have when attending a referral centre may not reflect those of owners visiting a first opinion centre; hence, a multi‐centre study is warranted to elucidate these differences.

In conclusion, it is the purpose of the pre‐anaesthetic consultation to convey information to pet owners and to provide an opportunity for pet owners to raise their questions and concerns. This study offers insight into the factors that concern owners regarding GA along with the level of knowledge that owners may be expected to possess ahead of these procedures. While anaesthetic mortality was found to be the leading category of concern, it can be appreciated that numerous other factors play a part in fuelling such anxiety. Provision of published mortality statistics may be helpful to certain clients when offering reassurance. Efforts made to further understand how referral clients feel about GA will go on to assist in the creation of supporting resources while improving channels of communication and empathy between veterinary professionals and clients.

## Author contributions

**C. Adami:** Supervision; formal analysis; writing – review and editing; methodology; investigation. **A. Shuttleworth:** Conceptualization; methodology; data curation; writing – original draft; writing – review and editing.

## Conflict of interest

Dr Adami is an Associate Editor of the *Journal of Small Animal Practice* and co‐author of this article. Dr Adami was excluded from editorial decision‐making related to the acceptance of this article for publication. No other conflicts of interest are declared.

## Funding information

No funding was received for this study.

## AI disclosure statement

No Artificial Intelligence Generated Content (AIGC) tools were utilised in the development of this manuscript.

## Supporting information


**Data S1.** Supporting Information

## Data Availability

The data that support the findings of this study are available from the corresponding author upon reasonable request.
