# Supplementary material for: “Up to and including death”: understanding anaesthesia‐related anxiety among pet owners in a tertiary‐care setting
Source: J Small Anim Pract. 2026 Jul 11;67(11):1091–9. doi: 10.1111/jsap.70168 (PMC13728970; doi:10.1111/jsap.70168)
Supplement: Supplementary file 1 [file JSAP-67-1091-s001.docx]

Consent to Participate

Dear Pet Owner,

Thank you for your interest in this study (A Survey of Pet Owner Awareness and Attitudes Towards

Veterinary Anaesthesia), your time is very much appreciated.

**Study background**

Dogs & cats commonly undergo general anaesthesia in veterinary practice and preparing owners ahead of this is a common task for veterinary staff. We know a lot of owners can be anxious ahead of their pet’s general anaesthetic and supporting owners with decision-making & consent are key parts of the pre-anaesthetic consultation.

However, to date there is very little literature within the veterinary field relating to pet owners’ knowledge of anaesthesia and their concerns surrounding it. In order to better support pet owners, we need to first see veterinary anaesthesia from their perspective.

Objectives of the study

- To determine pet owners’ level of anxiety relating to veterinary anaesthesia
- To better understand pet owners’ level of knowledge regarding veterinary anaesthesia
- To ascertain what information owners would like to know
- To ascertain how they would like to receive this information

Benefits of the study

- Improved ability of veterinary professionals to prepare pet owners ahead of general anaesthesia
- A deeper understanding of what drives pet owner dissatisfaction surrounding general anaesthesia
- The creation of resources to better educate pet owners (in a format they will find useful)
- Enhanced awareness of informed consent for general anaesthesia

**Participating in the study**

Your responses will be collected via an online questionnaire which takes approximately 5-10 minutes to complete. If your pet has undergone multiple general anaesthetics, please consider the most recent event for the purposes of this questionnaire. Please note, this survey may only be completed by each participant once and the survey will close Saturday 31st May 2025.

Thank you and kind regards,

Adam Shuttleworth

Senior Clinical Training Scholar in Veterinary Anaesthesia

University of Cambridge

By completing this questionnaire, you agree to the following terms:

- Your participation in this study is entirely voluntary and you understand that you may withdraw from the study at any point
- Your responses to this questionnaire will be completely anonymous and no identifying data will be obtained
- There will be no incentive offered for respondents
- The anonymised responses that you provide may contribute to publication of this study

**Do you consent to participate in this study?**

- Yes
- No

Demographics

What is your age (years)?

- <20
- 20-29
- 30-39
- 40-49
- 50-59
- 60-69
- 70-79
- >80
- Prefer not to say

What is your gender?

- Male
- Female
- Non-binary
- Other
- Prefer not to say

What is your field of work? (If you are retired, please indicate your previous field of work)

- Agriculture
- Animal care (including veterinary medicine)
- Art & entertainment
- Education & academia
- Environment & energy
- Hospitality
- Human healthcare & social work
- Information technology
- Law
- Leisure & tourism
- Manufacturing & construction
- Media & marketing
- Public services
- Retail & sales
- Science (including pharmaceuticals & engineering)
- Transport
- Other (please specify)
- Not applicable
- Prefer not to say

What is your highest level of qualification?

- GCSE/O-level
- A-level, BTEC or equivalent
- Apprenticeship
- Foundation degree
- Bachelor’s degree (BSc or BA)
- Master’s degree (MSc or MA)
- Doctor of philosophy (PhD) or other doctorate
- Other (please specify)
- Prefer not to say

What species is/was your pet?

- Dog
- Cat

What was your pet’s state of health ahead of their anaesthetic?

- Healthy
- Mildly unwell - illness present but not impacting day-to-day life
- Moderately unwell - illness impacts day-to-day life
- Severely unwell - illness was a threat to life
- Unknown

Which service did you visit with your pet?

- Cardiology
- Dermatology
- Medicine
- Neurology
- Oncology
- Ophthalmology
- Orthopaedics
- Soft tissue surgery
- Unknown

What was the nature of your pet's procedure (please select the most relevant option)?

- Elective surgery (e.g. neutering) - this a planned surgery usually scheduled in advance
- Non-elective or emergency surgery
- Diagnostic investigations
- Non-surgical therapy (e.g. endoscopic foreign body removal)
- Unsure

What was the timeframe between you being recommended the procedure and the commencement of the procedure?

- Less than 24 hours
- Over 24 hours but less than 1 week
- Over 1 week but less than 1 month
- Over 1 month
- Unsure

Attitude Towards Anaesthesia

On a scale of 1-5, how would you describe your feeling towards your pet's anaesthetic in the run up to the procedure?

1. Terrified
2. Anxious
3. Indifferent
4. Excited

Did you have any concerns ahead of your pet's anaesthetic?

- Yes
- No

What were your concerns?

Had your pet undergone general anaesthesia (at any practice) before this procedure?

- Yes
- No
- Unsure

Awareness of Anaesthesia

When undergoing general anaesthesia at a referral centre, who do you think could perform you pet's anaesthetic (you may select multiple options)?

- A veterinary surgeon WITH advanced qualification(s) in anaesthesia
- A veterinary surgeon WITHOUT advanced qualification(s) in anaesthesia
- A registered veterinary nurse WITH advanced qualification(s) in anaesthesia
- A registered veterinary nurse WITHOUT advanced qualification(s) in anaesthesia
- Other (please specify)

What do you believe to be the risk of anaesthetic-related death in a healthy dog or cat? (Please give your answer in the form of a percentage)

- Dog:
- Cat:

What do you believe to be the risk of anaesthetic-related death in a healthy human? (Please give your answer in the form of a percentage)

Consent for Anaesthesia

Did you receive all of the information relating to anaesthesia that you wanted ahead of your pet’s general anaesthetic?

- Yes
- No

What further information would you have liked ahead of the procedure?

What would have been your preferred way to receive this information?

- Consultation with the person carrying out the procedure
- Consultation with the person carrying out the anaesthetic
- Leaflet/letter
- Webpage
- Video
- Other (please specify)

Did you have enough information ahead of your pet's anaesthetic to provide informed consent?

- Yes
- No
- Unsure

According to our most recent literature, the risk of anaesthetic-related death for a healthy dog is 0.009-0.05% and for a healthy cat the risk is 0.11%.

For pets that are unwell, the risk rises to 1.3-1.4%.

References:

- Brodbelt, D., 2009. Perioperative mortality in small animal anaesthesia. The Veterinary Journal, 182(2), pp.152-161
- Shoop-Worrall, S.J., O’neill, D.G., Viscasillas, J. and Brodbelt, D.C., 2022. Mortality related to general anaesthesia and sedation in dogs under UK primary veterinary care. Veterinary Anaesthesia and Analgesia, 49(5), pp.433-442

If you had been given the above percentages ahead of your pet's procedure, how would this have impacted your level of reassurance before they underwent general anaesthesia?

- Less reassured
- The same
- More reassured
